# Relationships between Caregiving Stress, Depression, and Self-Esteem in Family Caregivers of Adults with a Disability

**DOI:** 10.1155/2017/1686143

**Published:** 2017-10-17

**Authors:** DeokJu Kim

**Affiliations:** Department of Occupational Therapy, Health Science College, Cheongju University, Cheongju, Republic of Korea

## Abstract

This study aimed to examine the relationships between caregiving stress, depression, and self-esteem of family caregivers of an adult person with a disability and to identify their effects on their caregiving burden. The study was performed with 108 care providers of adult people with a disability who visited hospital rehabilitation centers. Caregiving stress showed a significant positive correlation with depression and with economic and psychological stress, and it showed a significant negative correlation with self-esteem. When the care provider was aged, female, and without a job and the caregiving cost and time were higher, the caregiving stress was high. When the care provider was female and had a lower income, the depression index was high. When the person with a disability was male and in the forties and the level of disability was higher, the caregiving stress was high. When the disability was related to spinal cord damage, the care provider's depression index was the highest. To reduce caregiving stress and depression in the family caregivers and to improve their self-esteem, continuous support and help from specialists are necessary. Additionally, a variety of intervention programs need to be designed to motivate them to participate regularly at the community level.

## 1. Introduction

According to surveys, the disabled population has continued to grow, with 3.09% in 2000, 4.59% in 2005, and 5.61% in 2011 [1]. A person with a disability often has no choice but to rely on his/her family. Supporting a family member with a disability is one of the most challenging experiences. Many preceding studies have reported that family caregivers of a person with a disability experience deterioration in mental and physical health due to stress [2, 3]. Addressing familial burdens arising from an increasing population of the elderly and of patients with chronic diseases, Goodman and Punoos [4] called family caregivers who care for a patient for a long period a “second victim” and the family of a person with a chronic disease a “potential patient.” Issues around the presence of disability within a family are not just limited to the person with the disability, but they concern the whole family as the mental and financial burden are shared and care is provided as per the level of severity of the disability [5].

Some families build a closer relationship and successfully look after the member with a disability using a variety of resources, while other families experience more tensions, conflicts, and frustrations between members [6]. Families who experience growing conflicts also experience sorrow, desperation, anger, and lowered self-esteem internally, and, on the outside, they experience physical burden, lack of time owing to work and caregiving, and financial challenges [7]. If these feelings and emotions are left unresolved, familial tensions and conflicts become chronic and extreme, which occasionally leads the family to fall apart [5]. At varying degrees, majority of the families with a member with a disability show depression symptoms and feel stress over their caregiving responsibilities. Caregiving stress is, in other words, expressed in the form of familial tensions and pressure, which is inevitably experienced owing to the imbalances between the caregiving responsibility and actual capability [8]. Families are put in a situation that is overwhelming and they experience mental and psychological confusion, which acts as a threat to their physical and mental health. Therefore, it is critical that the family members measure their level of stress and maintain their health.

Depression influences one's behavior, cognition, and emotions, and it significantly brings down the quality of care [9]. Depression is caused by the negative evaluation of oneself, which makes it difficult to carry out daily routines as one feels fatigued and loses motivation. It may also lead to physical pain and even suicidal behavior [10]. Hence, it is important to seek solutions to relieve such symptoms and to improve the health of family caregivers of a person with a disability to protect their mental health and to ensure that they lead a healthy family life.

Self-esteem is one of the characteristics that enable individuals to cope with hardships. Rosenberg [11] defined self-esteem as a subjective evaluation of oneself, and he proposed that a strong self-esteem indicates that one accepts and respects oneself and believes that he/she is a valuable human being. Coopersmith [12] defined self-esteem as a level of self-evaluation that is created and maintained by oneself, which is expressed either positively or negatively. It makes the individual see oneself as capable, important, successful, and valuable. He argued that self-esteem serves as a critical component in building a healthy mindset and personality, leading the individuals to self-realization. In other words, self-esteem, as a factor of self-concept, pertains to recognizing oneself as a valuable person. Self-esteem also mediates a variety of stressors experienced in daily life and serves as a social resource for a person with depression and as an internal resource that protects one from negative influences in a stressful environment [13]. In reality, however, families who support a member with a disability experience the lowering of self-esteem due to the difficulties they face. Low self-esteem creates a vicious cycle in which one is reluctant to ask for help and narrows one's social network, which in turn aggravate the burden of care. A person with a strong self-esteem, on the other hand, experiences less stress as he/she can protect himself/herself from psychological challenges and is able to respond to situations more positively [7]. Therefore, it is very important that we find ways to help the families improve self-esteem.

The studies that have been conducted on supporting a family member with a disability have largely focused on the difficulties and stress experienced by parents of a child with a disability. Very few studies have examined the stress, depression, and self-esteem of family caregivers of an adult with a disability. Therefore, this study aimed to examine the above, to analyze the correlations between the factors, and to find ways to improve the quality of life of family members caring for an adult with a disability. This study also sought to provide basic data necessary for designing interventions to mitigate depression and stress and to improve self-esteem.

## 2. Methods

### 2.1. Participants

The study subjects were family members of adult persons with a disability who visited rehabilitation centers at hospital S and A in S city, from June 1, 2016, to July 5, 2016. I briefed the subjects on the purpose and procedures of this study and requested their consent. The 115 family members who consented to participation in the study were questioned regarding the level of caregiving-related stress, depression, and self-esteem, and 108 questionnaires, excluding 7 invalid ones, were used for analysis. The unit and currency applied to the expression of the participants' monthly caregiving expenses and their average monthly income were Korean won.

### 2.2. Instruments

#### 2.2.1. Caregiving-Related Stress

We used the tools used by Kim (2010) to assess the burden of caregiving experienced by the family members, which was based on the Caregiver Burden Inventory developed by Novak and Guest [14] to measure the level of caregiving stress. The tool contains 20 questions across the following 4 areas: 5 questions on financial stress, 5 on physical stress, 5 on psychological stress, and 5 on social stress. Financial stress refers to the stress pertaining to the expenditure related to treatment. Physical stress included the experience of chronic fatigue and physical conditions. Psychological stress pertains to the negative feelings experienced related to caregiving. Social stress concerns the stress arising from conflicting social roles. A 5-point Likert scale is used, with 1 representing “not at all” and 5 indicating “very much so.” Total scores range from 25 to 125 points, with a higher score indicating a higher level of stress. Cronbach's *α* of this tool reported in previous studies was 0.86 [15].

#### 2.2.2. Depression

The level of depression was assessed using the Korean Depression Scale (KDS) developed by M. S. Lee and M. K. Lee [16]. This scale comprises 30 questions, with 5 questions for each of the following 6 areas: negative thoughts about the future, negative thoughts about oneself, anxiety, restlessness, depressive feelings, physical symptoms, and loss of motivation. Each question is rated on a 5-point Likert scale, with 0 representing “not at all” and 4 indicating “very much so.” The gross score ranges from 0 to 120, with a higher score indicating a higher level of depression. The tool's Cronbach *α* was 0.88 [16]. I evaluated the total score of KDS in this study.

#### 2.2.3. Self-Esteem

Rosenberg's Self-Esteem Scale (1985) was used. This scale is a self-report tool that includes 10 questions, with 5 positive and 5 negative statements. Responses are made on a 4-point Likert scale, with 1 representing “generally not” and 4 indicating “always.” Negative statements are reverse scored and the total scores range from 10 to 40, with a higher score indicating a higher level of self-esteem. The tool's Cronbach *α* was 0.83 [17]. I evaluated the total score of Rosenberg's Self-Esteem Scale in this study.

### 2.3. Analysis Methods

This study used the SPSS 18.0 statistics program for the analysis.* t*-test and ANOVA were carried out to evaluate associations of caregiving stress, depression, and self-esteem levels with demographic and personal characteristics of the subjects, and the Duncan group categorization was used as a post hoc test. Correlations between caregiving stress, depression, and self-esteem were examined using the Pearson correlation coefficient and the variables affecting caregiving stress were examined using a multiple regression analysis. The significance level is *α* = 0.05.

## 3. Results

### 3.1. General Characteristics of the Subjects

#### 3.1.1. General Characteristics of the Family Caregivers

The family caregivers showed the following characteristics: the female group (65.8%) was larger than the male group was (34.2%); the distribution across age groups was as follows: 60 years or older (38.1%), 50 to 59 years (33.3%), and 40 to 49 years (15.7%); the most commonly observed relationship with the person with a disability was spouse (62.1%); the largest group had no occupation (66.7%); the distribution according to academic qualifications was as follows: Bachelor's degree (37.9%), high school graduate (37.0%), and middle school graduate or lower (18.5%); 81.5% of them were married; the distribution of monthly expenses incurred on supporting the person with a disability was as follows: 500,000*₩* to 1,000,000*₩* (36.1%) and 1,500,000*₩* to 2,000,000*₩* (26.4%); 64.9% of the participants spent 10 hours or longer each day on caregiving; and their monthly income distribution was as follows: 2,000,000*₩* to 3,000,000*₩* (25.9%), 4,000,000*₩* or more (24.1%), and 1,000,000*₩* to 2,000,000*₩* (22.2%) (Table 1).

#### 3.1.2. General Characteristics of the Persons with a Disability

The general characteristics of the adults with a disability were as follows: the 70 years or older age group was the largest (35.9%); the most common type of disability was brain stroke (63.3%), followed by spinal cord injury (17.3%), traumatic brain injury (10.2%), and other bodily injuries (9.2%); the distribution as per the grade of the disability was as follows: Grade 1 (50.0%), Grade 3 or higher (29.6%), and Grade 2 (20.4%) (in the disability grades, Grade 1 indicates the severest degree of disability, and the degree of disability decreases towards Grade 3); and the distribution as per duration of disability was as follows: less than 1 year (44.9%), 1 to 3 years (26.9%), and 10 years or longer (14.3%) (Table 2).

### 3.2. Correlation among Caregiving Stress, Depression, and Self-Esteem

The correlation between the caregiving stress, depression, and self-esteem experienced by the family caregivers was as follows: there was a significant correlation between the total score on caregiving stress and depression (*p* < 0.01). Additionally, a significant correlation was observed with the subareas of stress: financial stress (*p* < 0.01), physical stress (*p* < 0.01), and psychological stress (*p* < 0.01). No significance was observed for the correlation with social stress (*p* > 0.01). Financial stress (*p* < 0.01) and psychological stress (*p* < 0.01) showed a significant negative correlation with self-esteem. Finally, a significant negative correlation was observed between depression and self-esteem (*p* < 0.01) (Table 3).

### 3.3. Caregiving Stress, Depression, and Self-Esteem by the General Characteristics of the Subjects

#### 3.3.1. Associations of Demographic Characteristics with Study Measures for Caregivers

Associations of caregiving stress, depression, and self-esteem varied according to the general characteristics of the family caregivers. Specifically, significant differences were observed in the level of caregiving stress experienced according to sex (*p* < 0.01), age (*p* < 0.01), relationship with the patient (*p* < 0.01), marital status (*p* < 0.05), presence of occupation (*p* < 0.01), highest academic degree (*p* < 0.01), support expense (*p* < 0.05), caregiving hours (*p* < 0.01), and monthly income (*p* < 0.05). Further, significant differences were shown in the level of depression based on sex (*p* < 0.05), support expense (*p* < 0.01), and monthly income (*p* < 0.01). With reference to self-esteem, age (*p* < 0.01), highest education (*p* < 0.01), and monthly income (*p* < 0.01) resulted in significant differences. Thus, caregivers who were female, were aged 60 years or higher, were the care recipient's spouse, were single, had no occupation, were responsible for a high level of support expense, or spent more time on caregiving showed a higher level of caregiving stress. Similarly, among the female group, those who incurred higher support expenses or had a lower monthly income showed a higher level of depression. Finally, those who were younger, had a higher educational level, or had a higher monthly income showed a higher level of self-esteem (Table 4).

#### 3.3.2. Associations of Demographic Characteristics with Study Measures for Patients with a Disability

Associations of caregiving stress, depression, and self-esteem experienced by the family caregivers varied according to the general characteristics of the person they cared for. Specifically, caregiving stress differed significantly in terms of sex (*p* < 0.01), age (*p* < 0.01), grade of disability (*p* < 0.05), and duration of disability (*p* < 0.05). In terms of depression, age (*p* < 0.01), type of disability (*p* < 0.01), grade of disability (*p* < 0.01), and duration of disability (*p* < 0.01) resulted in significant differences. With reference to self-esteem, grade of disability (*p* < 0.01) and duration of disability (*p* < 0.01) led to significant differences. This indicated that, among the caregivers who catered to a male care recipient, a higher level of stress was shown when the latter was aged 40 to 49 years, when the grade of disability was higher, and when the duration of disability was longer. A higher level of depression was displayed among family caregivers when the care recipient was 40 to 49 years, the type of disability was stroke or spinal cord injury, the grade of disability was higher, or the duration of disability is less than a year. When the grade of disability was higher or the duration of disability was longer, the family members' self-esteem was lower (Table 5).

### 3.4. Factors That Influence Caregiving Stress

To identify the factors that influence caregiving stress, we conducted a multiple regression analysis with caregiving stress as the dependent variable (using the total caregiving stress score) and the total depression and self-esteem as the independent variables. In addition, I conducted a multiple regression analysis with “age, gender, whether they are currently employed, caregiving expenses, time spent on caregiving, and monthly income,” which can represent the general characteristics of caregivers, as independent variables.

Testing for multicollinearity between the independent variables showed that none of the Variance Inflation Factors crossed 2.5, indicating no multicollinearity. The variable that exerts the greatest influence on caregiving stress was the total depression score (*β* = 0.445) followed by self-esteem (*β* = −0.293), age among general characteristics (*β* = 0.515), time spent on caregiving (*β* = 0.401), and caregiving expenses (*β* = 0.303) (Table 6).

## 4. Discussion

This study was designed to examine the relationships between the caregiving stress, depression, and self-esteem experienced by family caregivers of an adult patient with a disability and to understand the factors influencing caregiving stress to help relieve the burden on the caregivers.

The analysis of the correlations between caregiving stress, depression, and self-esteem revealed that caregiving stress had a positive correlation with depression and that financial and psychological stress (subitems of caregiving stress) showed a significant negative correlation with self-esteem. Further, depression and self-esteem were negatively correlated. This indicates that though family caregivers may have accepted the presence of a disability to a certain degree, they continued to experience severe stress owing to the irrecoverable state of the disability and the care recipient's continuous need for care. This psychological burden led to loss of self-esteem, finally resulting in depression [18]. Thus, caregivers need to seek professional help to handle their stress, but there are few programs or professionals who can help relieve such stress. Therefore, it is necessary that local community centers implement intervention programs for families, to help them reduce such stress. Of most common interventional activities for caregiving stress, a group consultation program, which had been found to be widely effective, can address the caregivers' emotional needs, can be used to teach behavioral and relaxation techniques, and can act as a source of social support. Such a program can help the caregivers talk about their feelings with others and feel less isolated, which will help reduce the stress and will help participants separate themselves from stress [19, 20]. Regular participation in such programs will improve caregivers' self-esteem and reduce depression.

The varying degrees of caregiving stress, depression, and self-esteem with reference to the general characteristics of the subjects showed that female caregivers, who are older, are the care recipient's spouse, have no occupation, and spend more time and money on caregiving showed a higher level of stress. Additionally, female caregivers who spent more on caregiving and earned a lower income showed a higher level of depression, while caregivers who were younger, had a higher educational level, and had higher income showed a higher level of self-esteem. Sim and An's study [21] results also showed that a caregiver who incurs higher expenditure on caregiving and has a lower income showed a lower level of caregiving stress, which is in line with this study's findings. If a caregiver is financially vulnerable, one has to spend most of the income on caregiving and cannot afford to save for the future or enjoy leisure, and his/her quality of life will be hampered. A female caregiver who has no occupation naturally experiences more difficulties because of having to make ends meet, which could lead to depressive feelings. These findings show that an aged spousal caregiver finds caregiving more stressful than others do. As medical advances have continued to extend the life span of people with disabilities, the strongest fear for a caregiver who becomes aged is that he/she herself is getting older [22]. Additionally, an elderly caregiver would fear that when he/she dies, there will be no one left to take care of the person with a disability [23]. Spousal conflict with a care recipient who is the caregiver's husband is one of the factors that aggravates caregiving stress or depression. The wife feels that her stress over caregiving is not appreciated by the other family members and becomes depressed; she considers divorce but soon gives up because she is not financially independent; and she goes back to caregiving. This leads to a vicious cycle, and the relationship deteriorates with no communication between the couple [24]. Such deepened divides between couples worsen the family atmosphere.

With reference to the general characteristics of the care recipient, caregiving stress was strong when the care recipient was male, was aged 40 to 49 years, or had a higher grade of disability. When the patient is aged 40 to 49 years, a higher grade of disability led to a higher level of depression in the caregiver, and having a disability owing to a spinal cord injury was related to a higher level of depression. The more severe the disability was, the stronger the caregiving stress was. This may be because a person with a severe disability would have to rely more on the caregiver, meaning that the caregiver will have less time to spend on oneself and would face more difficulties and stress. Any family member with a relative who had a physical disability may experience depression, but it is notable that the caregivers of persons with spinal cord injury showed a high level of depression. In a study on patients with spinal cord injury, Tatiana [25] found that 50.6% of the respondents reported that their spousal relationship deteriorated after the disability occurred, indicating that the injury has a serious effect on the spousal relationship. In particular, loss of sexual functionality greatly undermines satisfaction with life, and 60% of the persons with spinal cord injury said they had issues with sex life and that the sexual dysfunction may have caused high stress in their spouse [3]. Those who experienced spinal cord injury at a young age and those who suddenly lost a source of income with no time for preparation experienced great pressure both financially and mentally.

Support programs for people with disabilities in Korea are largely focused on individual support and services for people with a disability, and no support is provided to their family [26]. It is important that measures are taken to provide specific and realistic assistance to family members who are financially vulnerable. Additionally, community service institutions for those with a disability should develop programs that invite people with a disability and their spouses together, to help them build a good relationship. However, such programs should not be temporary, and they need to be provided in a systematic manner, offering psychological and social support.

Depression was found to be a factor affecting caregiving stress. Depression causes caregivers to lose the will to live, tire easily, and feel physically heavy accompanied by physical pain [27]. Caregivers who take care of family members with disabilities frequently complain of constant pain in limbs and back as they do not have time to take care of themselves [21]. The physical pain triggers psychological burden and the symptom of depression, leading to demotivation. Depression of caregivers eventually aggravates caregiving stress.

Self-esteem was also identified as a factor that influences caregiving stress. A caregiver with strong self-esteem, that is, one who sees oneself as valuable and positive, is able to face the painful and challenging burden arising from caring for a person with a disability in a positive way, which will lead to a lower level of stress. Therefore, family caregivers need to be provided with psychological support to help them accept themselves as valuable and to help them deal with issues positively. It is necessary to facilitate the realization of positive experiences that can be derived from caregiving in addition to the negative experiences [28]. This may lessen the burden of support and help them accept the responsibility while having a positive image of themselves and feeling awarded as a more mature person [29]. The factors exerting the greatest influence in caregiving stress out of general characteristics were age, time spent on caregiving, and caregiving expenses, in that order. Older people are more prone to muscular skeletal disease. Symptom severity is likely to be worse for caregivers who provide physical care for family members with disabilities and could work as a factor aggravating caregiving stress. The higher the proportion of caregiving expenses to total income is and the longer the time of caregiving is, the harder it is to invest time and money in leisure for rest and recreation (Sim, 2013). Leisure enhances the level of satisfaction with life and ability to deal with stress, thereby playing an important role in self-realization and decent quality of life. If leisure activities that are highly correlated with quality of life are cut off from the lives of caregivers because of the burden of caregiving expenses and increase in the time spent on caregiving, it would result in more caregiving stress [30].

South Koreans are strongly family oriented and the responsibility of looking after a patient with a disability often falls upon the family. In this sense, a family can only be happy when they are considered physically and psychologically stable for providing care. For healthy families and the society, practical policies need to be prepared to lend financial and emotional help not only to those with a disability but also to their family caregivers.

The limitation of this study was that the subjects were visitors of particular hospitals located in Seoul, and therefore they do not reflect the overall characteristics of family caregivers across all regions. Future studies should ensure that subjects cover a wider area and the findings are more representative of the general public.

## 5. Conclusions

This study sought to examine the relations between caregiving stress, depression, and self-esteem experienced by the family members of an adult patient with a disability and to identify the factors influencing caregiving stress. Caregiving-related stress showed a significant correlation with depression, and financial and psychological stress, subitems of caregiving stress, had a significant negative correlation with self-esteem. Caregiving stress was stronger among caregivers who were female, older, and the spouse of the care recipient; had no occupation; and spent more time and money on caregiving. Depression level was higher in those who were female and had a lower income.

Caregiving stress was higher among those whose care recipient was male and aged 40 to 49 years, while depression levels were higher among those whose care recipient suffered from spinal cord injury. In order to reduce the levels of caregivers' stress and depression and to improve their self-esteem, continued and consistent support and help from professionals are needed. A variety of intervention programs need to be planned and implemented for family caregivers at the national and municipal levels.

## Figures and Tables

**Table 1:** General characteristics of family caregivers (*N* = 108).

Classification	Number (*N*)	Percent (%)
Sex		
Male	37	34.2
Female	71	65.8
Age		
30~39	14	12.9
40~49	17	15.7
50~59	36	33.3
60 or older	41	38.1
Relationship with patient		
Spouse	67	62.1
Child	32	29.6
Others	9	8.3
Whether they are currently employed		
Yes	36	33.3
No	72	66.7
Highest academic education		
Midschool graduated or lower	20	18.5
High school graduate	40	37.0
Bachelor's degree	41	37.9
Master's or higher	7	6.6
Marital status		
Married	88	81.5
Single	20	18.5
Caregiving expense (*₩* 10,000)		
Less than 50	5	4.6
50 to 100	39	36.1
100 to 150	23	21.3
150 to 200	30	26.4
200 or more	11	11.6
Time spent on caregiving (per days)		
Less than 5 hrs	24	22.2
5 to 10 hrs	14	12.9
10 or more hrs	70	64.9
Monthly income (*₩* 10,000)		
Less than 100	13	12.1
100 to 200	24	22.2
200 to 300	28	25.9
300 to 400	17	15.7
400 or more	26	24.1

**Table 2:** General characteristics of adults with a disability (*N* = 108).

Classification	Number (*N*)	Percent (%)
Sex		
Male	51	47.2
Female	57	52.7
Age		
40 or younger	16	14.8
40~49	14	12.9
50~59	16	14.8
60~69	25	23.1
70 or older	37	34.4
Highest academic education		
Midschool graduated or lower	12	11.1
High school graduate	51	47.2
Bachelor's degree	36	33.3
Master's or higher	9	8.4
Marital status		
Married	77	71.2
Single	13	12.1
Divorced/separated/widowed	18	16.7
Type of disability		
Stroke	65	60.2
Traumatic brain injury	13	12.0
Spinal cord injury	20	18.5
Other bodily injuries	10	9.3
Grade of disability		
Grade 1	52	48.1
Grade 2	23	21.3
Grade 3 or higher	33	30.6
Period of disability		
Less than a year	46	42.5
1 to 3 years	28	25.9
3 to 6 years	6	5.5
6 to 10 years	12	11.1
10 years or longer	16	15.0

**Table 3:** Correlation between caregiving stress, depression, and self-esteem (*N* = 108).

	Total caregiving stress	Financial stress	Physical stress	Psychological stress	Social stress	Depression	Self-esteem
Total caregiving stress	1	0.786^*∗∗*^	0.883^*∗∗*^	0.719^*∗∗*^	0.589^*∗∗*^	0.445^*∗∗*^	−0.136
Financial stress		1	0.699^*∗∗*^	0.297^*∗∗*^	0.153	0.362^*∗∗*^	−0.336^*∗∗*^
Physical stress			1	0.473^*∗∗*^	0.367^*∗∗*^	0.382^*∗∗*^	−0.171
Psychological stress				1	0.512^*∗∗*^	0.349^*∗∗*^	−0.473^*∗∗*^
Social stress					1	0.220	−0.100
Depression						1	−0.570^*∗∗*^
Self-esteem							1

^*∗∗*^ *p* < 0.01.

**Table 4:** Caregiving stress, depression, and self-esteem according to the general characteristics of the subjects (*N* = 108).

Variables	Caregiving stress		Depression		Self-esteem	
	^a^M ± SD	*p*	M ± SD	*p*	M ± SD	*p*
Sex						
Male	62.69 ± 12.63	0.009^*∗∗*^	44.76 ± 15.79	0.041^*∗*^	27.76 ± 4.31	0.988
Female	71.55 ± 14.33		54.25 ± 30.10		27.78 ± 5.49	
Age						
30~39	54.75 ± 16.51	0.001^*∗∗*^	46.00 ± 21.81	0.782	32.20 ± 6.61	0.002^*∗∗*^
40~49	67.80 ± 7.92		53.00 ± 29.63		29.03 ± 2.47	
50~59	65.86 ± 14.47		51.93 ± 27.71		24.25 ± 2.31	
60 or older	75.00 ± 12.19		45.96 ± 25.61		26.22 ± 5.87	
Relationship with patient						
Spouse	71.98 ± 15.11	0.008^*∗∗*^	51.63 ± 26.52	0.176	28.05 ± 5.34	0.576
Child	64.44 ± 10.83		48.88 ± 29.60		27.77 ± 5.54	
Other	56.60 ± 11.06		49.08 ± 26.28		26.00 ± 5.10	
Marital status						
Married	67.37 ± 14.61	0.041^*∗*^	47.34 ± 25.67	0.172	27.80 ± 5.21	0.306
Single	75.33 ± 10.88		58.66 ± 28.67		27.66 ± 4.69	
Whether they are currently employed						
Yes	63.21 ± 13.15	0.001^*∗∗*^	47.74 ± 22.01	0.082	27.81 ± 14.10	0.924
No	81.47 ± 16.87		57.08 ± 133.06		27.69 ± 17.06	
Highest education						
Midschool or lower	83.28 ± 9.77	0.001^*∗∗*^	58.07 ± 34.59	0.053	25.64 ± 7.04	0.008^*∗∗*^
High school graduate	61.58 ± 11.49		46.89 ± 21.85		26.24 ± 4.32	
Bachelor's degrees	70.61 ± 12.69		50.25 ± 25.61		30.03 ± 4.23	
Master's or higher	52.50 ± 12.12		24.50 ± 16.74		29.00 ± 1.15	
Caregiving expense (*₩* 10,000)						
Less than 50	57.00 ± 16.06	0.015^*∗*^	58.64 ± 14.06	0.001^*∗∗*^	20.00 ± 4.12	0.089
50 to 100	65.55 ± 14.42		52.62 ± 24.13		28.41 ± 4.46	
100 to 150	64.88 ± 10.19		44.63 ± 14.82		29.35 ± 5.14	
150 to 200	71.86 ± 16.31		47.13 ± 23.06		26.60 ± 5.67	
200 or more	82.85 ± 12.67		82.00 ± 33.67		27.42 ± 4.27	
Time spent on caregiving						
Less than 5 hrs	56.50 ± 16.00	0.005^*∗∗*^	51.06 ± 22.86	0.815	28.86 ± 5.33	0.663
5 to 10 hrs	63.73 ± 9.52		43.75 ± 24.28		27.50 ± 4.17	
10 or more hrs	71.69 ± 14.08		49.32 ± 27.69		27.52 ± 5.20	
Monthly income (*₩* 10,000)						
Less than 100	81.82 ± 11.90	0.012^*∗*^	67.53 ± 21.01	0.001^*∗∗*^	29.00 ± 5.74	0.002^*∗∗*^
100 to 200	71.80 ± 10.97		36.44 ± 19.43		23.46 ± 5.55	
200 to 300	67.90 ± 15.60		49.90 ± 27.01		27.66 ± 5.07	
300 to 400	68.00 ± 18.61		54.83 ± 23.49		28.41 ± 3.44	
400 or more	61.85 ± 9.49		31.52 ± 7.19		30.09 ± 3.57	

^*∗*^ *p* < 0.05, ^*∗∗*^*p* < 0.01, and ^a^M ± SD are the comprehensive points of evaluation tool.

**Table 5:** Caregiving stress, depression, and self-esteem according to the general characteristics of the person with a disability (*N* = 108).

Variables	Caregiving stress		Depression		Self-esteem	
	^a^M ± SD	*p*	M ± SD	*p*	M ± SD	*p*
Sex						
Male	74.19 ± 12.84	0.001^*∗∗*^	47.08 ± 24.95	0.536	28.02 ± 5.65	0.679
Female	63.80 ± 13.94		50.80 ± 27.54		27.57 ± 4.63	
Age						
40 or younger	65.66 ± 13.94	0.002^*∗∗*^	58.33 ± 37.41	0.003^*∗∗*^	25.33 ± 8.94	0.072
40~49	82.50 ± 5.19		85.00 ± 31.17		30.50 ± 6.35	
50~59	80.40 ± 10.78		28.40 ± 10.84		30.60 ± 2.95	
60~69	70.10 ± 12.67		48.68 ± 18.54		28.00 ± 6.05	
70 or older	63.54 ± 14.15		47.87 ± 23.11		27.45 ± 4.46	
Type of disability						
Stroke	68.08 ± 13.45	0.061	52.28 ± 15.07	0.005^*∗∗*^	27.61 ± 4.80	0.056
Traumatic brain injury	75.14 ± 12.68		25.50 ± 10.15		29.00 ± 4.56	
Spinal cord injury	76.00 ± 11.75		55.73 ± 28.42		26.42 ± 5.38	
Other bodily injuries	74.71 ± 16.92		37.71 ± 8.87		27.92 ± 4.28	
Grade of disability						
Grade 1	76.60 ± 14.24	0.045^*∗*^	72.60 ± 19.71	0.001^*∗∗*^	18.80 ± 3.83	0.001^*∗∗*^
Grade 2	67.07 ± 14.10		54.42 ± 26.34		27.50 ± 4.34	
Grade 3 or higher	67.37 ± 13.63		49.00 ± 22.23		27.87 ± 4.27	
Period of disability						
Less than a year	64.78 ± 13.51	0.019^*∗*^	67.33 ± 31.32	0.001^*∗∗*^	29.06 ± 5.32	0.001^*∗∗*^
1 to 3 years	68.76 ± 17.07		38.12 ± 24.23		27.04 ± 4.45	
3 to 6 years	76.00 ± 12.06		63.00 ± 20.06		26.00 ± 3.26	
6 to 10 years	77.06 ± 12.78		55.55 ± 19.62		16.33 ± 2.64	
10 years or longer	76.50 ± 6.86		50.00 ± 8.73		25.53 ± 3.14	

^*∗*^ *p* < 0.05, ^*∗∗*^*p* < 0.01, and ^a^M ± SD are the comprehensive points of evaluation tool.

**Table 6:** Factors that influence caregiving stress (*N* = 108).

Independent variable	Nonstandardized coefficient		Standardized coefficient	*t*	*p*	95% confidence interval
	B	S.E	*β*			
Depression	0.243	0.056	0.445	4.332	0.001^*∗∗*^	0.146–0.340
Self-esteem	−1.251	0.322	−0.293	2.996	0.003^*∗∗*^	0.503–0.612
Age	8.773	1.449	0.515	9.054	0.001^*∗∗*^	3.001–8.337
Gender	4.341	2.679	0.124	1.224	0.136	0.593–5.411
Whether they are currently employed	4.719	4.401	0.140	1.072	0.287	0.300–1.258
Caregiving expense	4.535	1.023	0.303	4.432	0.001^*∗∗*^	2.005–7.038
Time spent on caregiving	8.437	1.988	0.401	4.243	0.001^*∗∗*^	1.366–8.086
Monthly income	4.401	3.554	0.136	1.238	0.219	0.297–2.423

Dependent variable: total score on caregiving stress. *R*^2^ = .710, adjusted *R*^2^ = .688, *F* = 32.209, and ^*∗∗*^*p* < 0.01.
